# Supportive supervision of nurses and its impact on healthcare access in sub-Saharan Africa

**DOI:** 10.4102/hsag.v30i0.3094

**Published:** 2025-11-07

**Authors:** Maserapelo G. Serapelwane, Gaotswake P. Kovane, Nokwanda E. Bam

**Affiliations:** 1 School of Nursing, Faculty of Health Sciences, North-West University, Mahikeng, South Africa

**Keywords:** access, healthcare, impact, nurses, nurse managers, supportive supervision

## Abstract

**Background:**

Supportive supervision of nurses remains a concern globally, particularly in sub-Saharan countries. It involves providing guidance, resources and encouragement to nurses to deliver high-quality patient care.

**Aim:**

This study aimed to systematically review literature regarding supportive supervision of nurses and its impact on healthcare access.

**Method:**

The study used a systematic review method, guided by the PICOT (Patient/Population, Intervention, Comparison, Outcome and Timeframe), which framed the review question. The systematic search was based on articles written in English from 2013 to 2023. Databases used for locating the articles were: Web of Science, Google Scholar, Scopus, EBSCOhost, SA ePublication, PubMed, Medline, PubMed and Science Direct. The Preferred Reporting Items for Systematic Reviews and Meta-Analyses (PRISMA) checklist was used to report the identified studies, while the quality of studies meeting the review criteria was assessed using the 11 questions from the Joanna Briggs Institute (JBI) critical appraisal checklist.

**Results:**

A total of 274 articles were identified, and data were extracted from five articles that met eligibility criteria. The study showed that supportive supervision of nurses improves access to healthcare by boosting job satisfaction and clinical competency. The negative impact of the lack of supportive supervision of nurses was related to declined performance because of a lack of policies or guidelines and dictatorial supervision.

**Conclusion:**

It is evident from the findings that supportive supervision of nurses enhances access to healthcare, whereas its absence negatively affects it.

**Contribution:**

The study provided new insights into how supportive supervision of nurses influences access to healthcare.

## Introduction

Supportive supervision enables nursing staff to enhance their performance in delivering healthcare services (World Health Organization [WHO] 2020). Access to healthcare is defined as the ability to obtain timely and appropriate services that align with individual needs, relative to the extent of those needs (Núñez, Sreeganga & Ramaprasad 2021). The current study systematically reviewed the existing literature on supportive supervision of nurses and its impact on access to healthcare.

In the Western United States (US) and Europe, a lack of supportive supervision of nurses resulted in failures to provide treatment for clients on chronic medication and difficulties in completing tasks during night shifts (Dall’Ora et al. 2020; Kluwer 2022). In the US, clients who missed their chronic medication at health facilities were hesitant to return for follow-up care and assessments (Kluwer 2022). Nurses who did not complete tasks during the night shift in the critical care unit experienced burnout because of a shortage of staff and equipment. These nurses did not receive clear guidance from their supervisors on how to overcome challenges they were facing (Goyal et al. 2021). The findings of studies conducted by Kluwer (2022) and Goyal et al. (2021) suggest that unsupportive supervision of nurses exerts a negative impact on the accessibility and provision of healthcare to clients and patients. A study conducted in Ireland by O’Shea et al. (2019) found that supportive supervision yields benefits, including motivated staff, improved access to healthcare services and reduced turnover. Inspired by these findings, the current study investigates the impact of supportive supervision on access to healthcare, potentially yielding similar benefits.

A study conducted in Saudi Arabia (Western Asia) found that nurses who lacked support and fair treatment from senior management in primary health facilities experienced higher levels of burnout and job dissatisfaction (Almadani 2019). Similarly, research done in Egypt by Mahmoud and El Saeed (2021) highlighted the absence of supportive supervision, characterised by abusive practices, which contributed to high turnover and absenteeism. These findings from Saudi Arabia and Egypt indicate that inadequate supervision, unequal treatment and mistreatment of nurses reflect a broader lack of supportive supervision by senior nursing managers across both hospital and primary healthcare settings (Almadani 2019; Mahmoud & El Saeed 2021). In addition, a study done in Malawi reported weak implementation of malaria policies because of insufficient supportive supervision following nurse training. Mwendera et al. (2019) emphasised the importance of ongoing supportive supervision after training to enhance adherence to policies and guidelines, and ultimately to improve quality care.

In Nigeria, one of the causes of stress and anxiety among nursing staff was identified as the lack of supportive supervision in the management of critically ill patients. Nursing staff in Nigeria further reported that their poor relationship with nursing managers led to their inability to seek support regarding training on how to operate the specialised equipment (Faremia et al. 2019). Studies conducted in some provinces of South Africa demonstrated a phenomenon of nurses being blamed for poor facility performance, fault-finding and the lack of management support while working in maternity units (Matlala & Lumadi 2019; Raliphaswa, Luhalima & Netshandama 2020).

In addition, South African nurses felt devalued by their nursing managers during supervision sessions and mentioned the lack of support from management as the main reason for the migration of advanced midwives to other international countries (Matlala & Lumadi 2019; Raliphaswa et al. 2020). The studies further discovered that shortage of nurses, a lack of material resources and low salaries influenced a lack of supportive supervision at both primary health facilities and hospital settings. A systematic review conducted in South Africa examined the impact of NIMART (Nurse-Initiated Management of Antiretroviral Therapy) training on human immunodeficiency virus (HIV) management but did not address supportive supervision or access to healthcare (Mbowenia & Makhado 2019). Unsupportive supervision of nurses is common in sub-Saharan Africa, yet its specific impact on healthcare access remains understudied (Almadani 2019; Mahmoud & El Saeed 2021). While previous research has used various methods, a comprehensive systematic review remains a palpable gap. This study addresses this knowledge gap by synthesising evidence regarding the impact of supportive supervision of nurses on access to healthcare in sub-Saharan Africa.

## Research methods and design

A systematic literature review approach was used to answer the research question (Higgins et al. 2024:13). This review was registered in the PROSPERO system, which aims to promote transparency and open science, with registration number CRD42023434240.The systematic review was carried out between 01 July and 31 August 2023. The methods section of the present study outlines the study objective, the steps taken to conduct the review, data collection and analysis procedures, as well as the results and discussion.

### Objective

The objective of this study was to examine supportive supervision of nurses and its impact on healthcare access in sub-Saharan Africa.

### Steps of systematic review

The study proceeded through four steps, namely: (1) framing of a focused review question, (2) searching the literature, (3) performing critical appraisal and (4) data extraction and synthesis, and summary of evidence (Higgins et al. 2024:29). The steps are discussed as follows:

#### Step 1: Framing of a focused review question

The researchers formulated a review question within a specific area of practice, stated as follows: ‘What is the impact of supportive supervision of nurses on access to healthcare in sub-Saharan Africa?’ PICOT, which stands for Participants (P), Intervention (I), Comparative (C) Outcome (O), Time frame (T), was used to guide the review question (Higgins et al. 2024:30). The participants in this study were any category of nurses and nurse managers providing health services to clients. Intervention was supportive supervision, comparative was unsupportive supervision and the outcome was access to healthcare. The time frame was 2013–2023. This was designed to ensure the retrieval of the most up-to-date information on the impact of supportive supervision of nurses on healthcare access.

#### Step 2: Searching the literature

**FIGURE 1:**
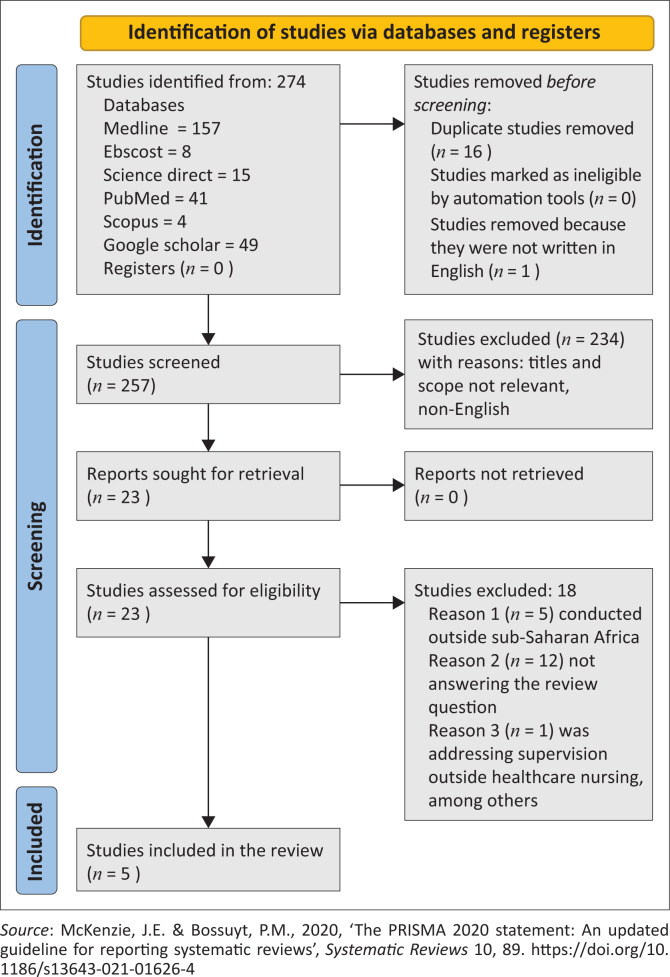
Identification of studies via databases and registers.

In this study, the researchers carried out a systematic literature search, with assistance from a librarian, to identify evidence relevant to the research question. (Higgins et al. 2024:67). The inclusion criteria encompassed all categories of nurses and nurse managers delivering services in sub-Saharan Africa, time frame was 2013 to 2023, only articles written in English were reviewed. The reason for selecting studies from 2013 to 2023 was to ensure manageable data and feasibility. The review considered qualitative, mixed-method, quantitative and systematic review articles. The researchers excluded the articles dwelling on different spatial locations other than sub-Saharan Africa. The following electronic databases were used for the literature search: Web of Science, Google Scholar, Scopus, EBSCOhost, SA ePublication, PubMed, Medline, PubMed and Science Direct. The keywords used for the literature search included impact, supportive supervision, nurses, nurse managers, access and sub-Saharan Africa. In addition, the researchers manually searched journals and the reference lists of other review articles to identify further relevant studies. The PRISMA checklist (McKenzie & Bossuyt 2020) was used to report the identified studies. Figure 1 illustrates the process of study identification.

#### Step 3 Critical appraisal

The quality of studies meeting the review criteria was assessed using the 11 critical appraisal questions from the Joanna Briggs Institute (JBI) critical appraisal checklist (The Joanna Briggs Institute 2017:3). The critical appraisal questions used are: (1) *Is the review question stated*? (2) *Were the inclusion criteria appropriate for the review question*? (3) *Was the search strategy appropriate*? (4) *Were the sources and resources used to search for studies adequate*? (5) *Were the criteria for appraising studies appropriate*? (6) *Was critical appraisal conducted by two or more reviewers independently*? (7) *Were there methods to minimise errors in data extraction*? (8) *Were the methods used to combine studies appropriate*? (9) *Was the likelihood of publication bias assessed*? (10) *Were recommendations for policy and/or practice supported by the reported data*? (11) *Were the specific directives for new research appropriate*? Consequently, three reviewers – M.G.S., G.P.K., and N.E.B. – assessed the eligibility of the retrieved articles. The article selection process proceeded as follows: (1) total identified articles: 274 (2); articles removed before screening: 17; (3) articles screened: 257; (4) articles excluded after screening: 234; (5) articles included in eligibility assessment: 23. Of these, 18 studies failed the inclusion criteria: five studies were conducted outside sub-Saharan Africa; 12 did not answer the review question and were unclear regarding the impact of supportive supervision on access to healthcare; and one study addressed supervision and access outside the context of healthcare and nursing. Consequently, the total number of articles included in the review was 5.

#### Step 4: Data extraction, synthesis and summary of evidence

Five studies, conducted in South Africa, Tanzania, Botswana, Mozambique and a sub-Saharan systematic review, met the eligibility criteria, and data were extracted. The extracted information included: author and publication year, country, population, study aim, findings and conclusion. The extracted data were analysed using thematic analysis (Clarke & Braun 2013). Table 1 presents a synthesis and summary of the evidence from the included studies.

**TABLE 1:** Synthesis and summary of evidence of included studies.

Author, year and country	Design	Population	Aim of the study	Findings from studies and conclusions
Nkomazana et al. 2016 Botswana	Qualitative research	Female nurses and nursing managers	To explore how district health managers can change their practice to create a more supportive environment for primary healthcare providers.	Dictatorial supervision demotivates healthcare workers, while supportive supervision has been shown to improve workforce performance, productivity, access to quality care, motivation and retention of staff.
Madede et al. 2017 Mozambique	Cluster controlled design-mixed methods	Nursing managers and registered nurses	To describe the impact of supportive supervision intervention on nurses’ turnover and job satisfaction.	Health facility access to maternal and neonatal care improved from below 50% after receiving supportive supervision. Nurses reported improvements to their performance because supervision motivated them to improve their approach to the management of women during labour. Supportive supervision made nurses realise that they work for the benefit of themselves as well as for the population they serve. Participants reported that unsupportive supervision may lead nurses to lose their job because of litigation, which may result in a decreased contribution to the health and well-being of the community at large.
Bailey et al. 2016 Sub-Saharan Africa	Systematic review	Published nursing supervision articles in the PHC facilities	To review the effects of supportive supervision on the quality of care, health worker motivation and performance.	Quality and access to health care improved after supportive supervision, evidenced by significant improvement in IMCI indicators, increased providers’ ability to offer a wide range of and improved access to integrated sexual reproductive health and HIV services/ART. Increased availability of treatment and adherence to protocols and guidelines. An increase in service accessibility and continuity of care to communities occurred after the implementation of supportive supervision.
Nyamhanga, Frumence & Hurtig 2021 Tanzania	Qualitative	District nursing managers, nursing managers at the facility level	To explore enablers of and barriers to supportive supervision in maternal newborn care at the district and hospital levels in Shinyanga region in Tanzania.	Firstly, ineffective supervision because of a lack of clear policies and guidelines for supportive supervision contributed to poor health service delivery. This was evident in poor maternal and newborn outcomes in one of Tanzania’s regions. Secondly, the lack of progress measurement in quality improvement because of shortfalls in setting goals and targets led to ineffective supervision.
Mhlongo & Lutge 2021 South Africa	A qualitative study	Facility managers	To explore and describe perceptions of facility managers regarding support and supervision of ward-based outreach teams in the national health insurance pilot teams	Supervision and support of team leaders by facility managers impacted positively on preventive and promotive health in rural communities by enhancing success in tracing of HIV treatment defaulters, health indicators for condom distribution improved and facility headcounts increased.

*Source:* Bailey et al. 2016; Madede et al. 2017; Nkomazana et al. 2016; Nyamhanga et al. 2021; Mhlongo & Lutge 2021

Note: Please see the full reference list of the article, https://doi.org/10.4102/hsag.v30i0.3094, for more information.

PHC, Primary Health Care; IMCI, integrated management of childhood illnesses; ART, Antiretroviral Therapy; HIV, human immunodeficiency virus.

### Ethical considerations

This article followed all ethical standards for research without direct contact with human or animal subjects.

## Results

Data were extracted from three qualitative studies, one systematic review and one cluster-controlled mixed-methods design. These five studies were conducted in countries within sub-Saharan Africa. The studies were written in English between 2013 and 2023. The categories of nurses represented in the selected studies include registered nurses and nursing managers from both primary healthcare facilities and hospitals. The studies aimed to: (1) explore how district health managers can change their practice to create a more supportive environment for primary healthcare providers; (2) describe the impact of supportive supervision intervention on nurses’ turnover and job satisfaction; (3) review the effects of supportive supervision on the quality of care, health worker motivation and performance; (4) explore enablers and barriers to supportive supervision in maternal and newborn care at district and hospital levels; (5) explore and describe perceptions of facility managers regarding support and supervision of ward-based outreach teams in the national health insurance pilot programmes. The findings and conclusions from the studies that met the inclusion and eligibility criteria were analysed using thematic data analysis (Clarke & Braun 2013).

Analysis of the studies highlighted two primary themes, sub-themes and sub-subthemes. The first theme reports factors related to the positive impact of supportive supervision of nurses on access to health care, while the second theme reports factors related to the negative impact of a lack of supportive supervision of nurses on access to health care.

Table 2 provides a summary of these themes and their related sub-themes.

**TABLE 2:** Supportive supervision of nurses and its impact on healthcare access in sub-Saharan Africa.

Theme	Sub-themes	Sub-subthemes
1. Factors related to the positive impact of supportive supervision of nurses on access to health care	1.1 Job satisfaction	1.1.1 Improved access to quality care 1.1.2 Work motivation 1.1.3 Realisation of benefits
	1.2 Clinical competency	1.2.1 Improved quality of primary health care priority programmes 1.2.2 Adherence to treatment protocols and guidelines 1.2.3 Availability of treatment
2. Factors related to the negative impact of the lack of supportive supervision of nurses on access to health care	2.1 Lack of clear policies and guidelines	2.1.1 Poor health outcomes 2.1.2 Lack of measurement of progress
	2.2 Dictatorial supervision	2.2.1 Demotivated staff

*Source:* Bailey et al. 2016; Madede et al. 2017; Nkomazana et al. 2016; Nyamhanga et al. 2021; Mhlongo & Lutge 2021

The analysis identified two main themes, which are presented in Table 2 and discussed as follows:

### Theme 1: Factors related to the positive impact of supportive supervision of nurses on access to health care

The factors related to the positive impact of supportive supervision yielded two sub-themes namely: (1) Job Satisfaction and (2) Clinical Competency.

The sub-theme ‘Job Satisfaction’ was further categorised into three sub-subthemes: (1) Improved access to quality care, (2) Work motivation and (3) Realisation of benefits.

Two studies conducted in Botswana and Mozambique verified that supportive supervision increased nurse motivation and made quality care more accessible (Madede et al. 2017; Nkomazana et al. 2016). Three studies established that primary health care facilities had improved access to quality care when nurses were provided with supportive supervision (Bailey et al. 2016; Madede et al. 2017; Nkomazana et al. 2016). The provision of quality care was evidenced by enhanced access to integrated sexual and reproductive health and ART services (Bailey et al. 2016).

In Mozambique, supportive supervision for registered nurses led to a significant increase in access to maternal and neonatal care, which had previously been below 50% (Madede et al. 2017). It is evident that supportive supervision made nurses realise they work for their benefit and the community they serve (Madede et al. 2017). In addition, supportive supervision enhanced work productivity and staff retention (Madede et al. 2017; Nkomazana et al. 2016). The findings demonstrate and confirm that job satisfaction emanates from supportive supervision that positively impacts access to healthcare in the health settings.

The sub-theme on clinical competency comprised three sub-subthemes: (1) improved quality of primary health care priority programmes, (2) adherence to treatment protocols and guidelines and (3) availability of treatment.

In this review, nurses stated that supportive supervision motivated them to increase their ability to provide care (Bailey et al. 2016) and improve their approaches in managing women during labour (Madede et al. 2017). Hence, an improvement in the quality of the maternal and neonatal health programme was reported (Madede et al. 2017). Supportive supervision also improved indicators for the integrated management of childhood illnesses (IMCI) (Bailey et al. 2016). In addition, supportive supervision of professional nurses led to success in tracing HIV treatment defaulters, health indicators for condom distribution improved and facility headcounts increased (Mhlongo & Lutge 2021). These demonstrate clinical competency that occurred after supportive supervision, which positively impacted access to maternal and neonatal care, IMCI, HIV treatment and condom use (Bailey et al. 2016; Madede et al. 2017). Furthermore, supportive supervision promoted adherence to protocols and guidelines and enhanced treatment availability (Bailey et al. 2016).

### Theme 2: Factors related to the negative impact of the lack of supportive supervision of nurses on access to health care

A lack of clear policies and guidelines, along with dictatorial supervision, were identified as sub-themes of factors related to the negative impact of the lack of supportive supervision of nurses on access to health care.

Poor health outcomes and a lack of measurement of progress emerged as sub-subthemes from a lack of clear policies and guidelines. In this regard, poor health outcomes were caused by ineffective supervision resulting from the absence of clear policies and guidelines for supportive supervision (Nyamhanga et al. 2021). This was manifested in poor maternal and newborn outcomes in one of the regions of Tanzania. The lack of progress measurement in quality improvement was because of no directives in setting goals and targets, consequently leading to ineffective supervision (Nyamhanga et al. 2021). Demotivated staff was a sub-theme that was a consequence of dictatorial supervision. A study conducted in Botswana established that dictatorial supervision led to demotivated staff, highlighting the negative impact of authoritarian leadership on employee morale (Nkomazana et al. 2016). It has also been reported that a lack of supportive supervision can result in litigation and job loss among nurses, which in turn contributes to staff demotivation (Madede et al. 2017).

## Discussion

The study findings highlighted factors demonstrating the positive impact of supportive supervision on access to health care, as well as factors reflecting the negative consequences of its absence. These are discussed as follows:

### Factors related to the positive impact of supportive supervision of nurses on access to health care

This review contends that a supportive supervision is crucial for nurse job satisfaction, which in turn enhances work performance and access to healthcare services. It is further confirmed that reproductive health services, antiretroviral treatment, and maternal and neonatal care have improved following supportive supervision. This finding aligns with a study conducted in East China that emphasises the importance of promoting supportive supervision to reduce job-related stress and staff turnover, ultimately improving job satisfaction and healthcare access (Lin et al. 2021). The findings in China on job satisfaction suggest that supportive supervision plays a key role in sustaining access to health care. Moreover, access to health care improves only when nurses receive supportive supervision. The current review reports that supportive supervision of nurses boosts work motivation, a key aspect of job satisfaction, eventually enhancing access to healthcare. According to Safari (2024), job satisfaction among nurses boosts performance and enables them to complete their duties more effectively. When nurses complete their duties effectively, patients are more likely to access the healthcare services they need.

The study verified that supportive supervision is associated with improved clinical competency in managing childhood illnesses and maternal and neonatal care. Furthermore, adherence to clinical protocols and guidelines was enhanced by supportive supervision. The finding demonstrates that the clients in the primary health care settings had access to the right treatment and care because of supportive supervision. In contrast to this review’s findings, a study conducted in Tajikistan, Asia, found that nurses’ misinformation about screening and diagnosis protocols at community level hindered access to hypertension management and treatment (Chukwuma et al. 2019). The finding by Chukwuma et al. (2019) suggests that failure to provide nurses with necessary information and protocols negatively affected access to healthcare. These further suggest a lack of supportive supervision, evident in the lack of knowledge about screening and diagnosis protocols interfering with access to healthcare. One of the key components of access to health care in health settings is the availability of resources to the clients (Kapse & Aurangabadkar 2020). Hence, this review found that supportive supervision for nurses led to increased treatment availability, highlighting the importance of supportive supervision in ensuring resource accessibility in primary healthcare.

### Factors related to the negative impact of the lack of supportive supervision of nurses on access to health care

This review shows that unclear policies and guidelines for supportive supervision have contributed to poor health outcomes in maternal and neonatal care. Similarly, Sabelo and Zuma (2025) found that unsupportive management and a lack of accessible protocols and guidelines resulted in poor quality care. In addition, Bangura et al. (2020) verified that unsupervised nurses exposed clients to a lack of essential information about potential vaccine side effects and follow-up vaccination schedules, limiting their access to crucial health information and immunisation services. Poor nursing outcomes often stem from insufficient supportive supervision of nurses, which in turn affects healthcare access (Sabelo & Zuma 2025). Consequently, the study done by Martin et al. (2021) highlights the need for organisations to invest in high-quality supervision practices to improve access to health care. This study confirms stagnation in quality improvement because of the absence of goal setting and targets, resulting in a lack of supportive supervision. A manifestation of poor health outcomes, a lack of goal setting and failure to set targets suggest a lack of supportive supervision that affects access to health care. Dictatorial supervision refers to a management or leadership style where the supervisor or manager exercises absolute control and authority (Nkomazana et al. 2016). According to Afolabi, Fernando and Bottiglieri (2018), poor management and a lack of supervisory support strongly demotivated nursing staff. This review reports that unsupportive supervision leads to nurse job loss because of litigation, ultimately reducing their contribution to community health and well-being. Dictatorial supervision leading to demotivated nurses reduces healthcare accessibility. This review suggests that authoritarian supervision, marked by strict demands for compliance and obedience, resulted in decreased motivation among nursing staff.

### Limitations

This review is limited by its inclusion and exclusion criteria, specifically focusing on sub-Saharan countries. The background of supportive supervision demonstrated notable benefits and its positive impact on access to healthcare in developed countries. Hence, the current review was confined to sub-Saharan contexts. The search terms used might have limited the scope, potentially excluding globally applicable studies. As a result, the findings may not be generalisable to global contexts, limiting the understanding of supportive supervision’s impact on healthcare access.

## Recommendations

### For nursing practice

Nursing managers should consider implementing supportive supervision as a management approach, given its demonstrated benefits in enhancing nurse performance and improving healthcare access. There should be regular implementation of continuous quality improvement and supportive supervision guidelines that are available in the Department of Health offices. Nurse managers should be educated about the differences between the supportive supervision approach versus the dictatorial management style. Debriefing sessions should be conducted for all nursing personnel to help reduce anxieties related to litigation and job loss.

### For nursing research

Development of guidelines for strengthening supportive supervision of nurses and access to healthcare is recommended. To conduct research focusing on the nurses’ views regarding the impact of supportive supervision on access to health care in one of the provinces of South Africa.

## Conclusion

The findings indicate that supportive supervision of nurses positively impacts access to healthcare by enhancing job satisfaction, which is reflected in work motivation and increased productivity. In addition, supportive supervision led to noticeable improvements in clinical competence, which manifested in improved quality of primary healthcare priority programmes, adherence to treatment protocols and guidelines, and availability of treatment. These improvements demonstrate that supportive supervision of nurses effectively increases access to healthcare. Factors related to the negative impact of the lack of supportive supervision of nurses on access to healthcare are evident in this study. This is reflected in the absence of clear policies and guidelines for supportive supervision, coupled with dictatorial supervisory styles. This confirms a lack of supportive supervision, which consequently contributes to demotivated nurses. The study clearly shows that demotivated nurses struggle to perform their duties effectively, ultimately hindering access to nursing care.
